# Prenatal care and child growth and schooling in four low- and medium-income countries

**DOI:** 10.1371/journal.pone.0171299

**Published:** 2017-02-03

**Authors:** Xiaoying Liu, Jere R. Behrman, Aryeh D. Stein, Linda S. Adair, Santosh K. Bhargava, Judith B. Borja, Mariangela Freitas da Silveira, Bernardo L. Horta, Reynaldo Martorell, Shane A. Norris, Linda M. Richter, Harshpal S. Sachdev

**Affiliations:** 1 Population Studies Center, University of Pennsylvania, Philadelphia, Pennsylvania, United States of America; 2 Hubert Department of Global Health, Rollins School of Public Health, Emory University, Atlanta, Georgia, United States of America; 3 University of the Witwatersrand / Medical Research Council Developmental Pathways to Health Research Unit, University of the Witwatersrand, Johannesburg, South Africa; 4 Department of Nutrition, University of North Carolina at Chapel Hill, United States of America; 5 Department of Pediatrics, S.L. Jain Hospital, Delhi, India; 6 USC-Office of Population Studies Foundation, Inc., and Department of Nutrition and Dietetics, University of San Carlos, Cebu City, Philippines; 7 Post-Graduation Program in Epidemiology, Faculty of Medicine, Federal University of Pelotas, Brazil; 8 University of the Witwatersrand, DST-NRF Centre of Excellence in Human Development, Johannesburg, South Africa; 9 Department of Pediatrics and Clinical Epidemiology, Sitaram Bhartia Institute of Science and Research, New Delhi, India; Centre Hospitalier Universitaire Vaudois, FRANCE

## Abstract

**Background:**

The effectiveness of prenatal care for improving birth and subsequent child outcomes in low-income countries remains controversial, with much of the evidence to date coming from high-income countries and focused on early-life outcomes. We examined associations between prenatal care visits and birth weight, height-for-age at 24 months and attained schooling in four low- and middle-income countries.

**Methods:**

We pooled data from prospective birth-cohort studies from Brazil, Guatemala, Philippines and South Africa. We created a prenatal care utilization index based on the number and timing of prenatal visits. Associations were examined between this index and birth weight, height-for-age at 24 months, and highest attained schooling grade until adulthood.

**Results:**

Among 7203 individuals in the analysis, 68.9% (Philippines) to 96.7% (South Africa) had at least one prenatal care visit, with most having at least four visits. Over 40% of Brazilians and Guatemalans had their first prenatal visit in the first trimester, but fewer Filipinos (13.9%) and South Africans (19.8%) did so. Prenatal care utilization was not significantly associated with birth weight (p>0.05 in pooled data). Each unit increase in the prenatal care utilization index was associated with 0.09 (95% CI 0.04 to 0.15) higher height-for-age z-score at 24 months and with 0.26 (95% CI 0.17 to 0.35) higher schooling grades attained. Although there was some heterogeneity and greater imprecision across sites, the results were qualitatively similar among the four different populations.

**Conclusions:**

While not related to birth weight, prenatal care utilization was associated with important outcomes later in life, specifically higher height-for-age at 24 months and higher attained school grades. These results suggest the relevance of prenatal care visits for human capital outcomes important over the lifecycle.

## Introduction

Prenatal care has long been advocated to improve maternal health and birth outcomes, and together with perinatal and postpartum care, was identified as a key instrument to reach targets such as reduced child mortality as part of Millennium Development Goals 4 and 5 [1], and continues to be a priority in the Sustainable Development Goals (Goal 3) [2]. Prenatal care has increased significantly in low- and middle-income countries (LMICs) in recent years, but over half of pregnant women in developing countries still do not receive the World Health Organization (WHO)-recommended minimum of four visits starting early in pregnancy [3]. Considerable efforts are underway, supported by WHO, the World Bank and others, to expand prenatal care in low- and middle-income countries [4,5].

Prenatal care has many dimensions, including the time of the initial visit, the number and spacing of visits, the services provided in each visit, the type of provider and provider setting, the assessment of risk status, the schedule of medical screening tests, and the inclusion of specific medical, educational, nutritional, and social support services [5]. The expected benefits of prenatal care visits depend very much on the contents of prenatal care. Routine checks including fetal heart auscultation, urinalysis, assessment of maternal weight, blood pressure, and fundal height allow close monitoring of fetal development and reduce birth risk factors. Screening activities such as genetic counseling and testing, ABO and Rh blood typing, and screening for anemia and for neural tube defects and aneuploidy can prevent or mitigate adverse birth outcomes [6]. Micronutrient supplementation has been found to have benefit for babies, especially in developing countries [7,8]. Prenatal care providers may provide consultation on breastfeeding, nutrition, smoking and drinking, drug use, and food safety, as well as support to relieve maternal stress. Finally, prenatal care offers entry points for a range of other programs—such as those related to nutrition, malaria, HIV and AIDS and TB—as well as for obstetric care [9]. Not all prenatal care providers include all this content, and little of this content is usually provided in LMICs.

Notwithstanding the potential benefits, increasing prevalence, and interest in prenatal care worldwide, the effectiveness of prenatal care is still debated. The high-income country (HIC) experience has been examined extensively, especially for the United States, which has higher preterm delivery and low birth weight (LBW) prevalence than most other high-income countries despite the high prevalence of prenatal care utilization [10–18]. The evidence from LMICs is limited, and mainly focuses on single countries, making cross-country comparisons difficult [19–23]. Moreover, most studies so far have focused only on neonatal outcomes [10,23], such as preterm birth and LBW. However, some important benefits may only appear in later life. For example, some micronutrients included in recommended prenatal vitamins are important for fetal brain development and for children's cognitive skills, but may not necessarily improve birth weight [24,25].

Better understanding of how prenatal care in LMICs relates to child outcomes would help improve public health policies. This study examined associations of prenatal care and three important dimensions of human capital, namely birth weight (BW) [26–31], height-for-age z-score (HAZ) at 24 months [32–38], and school attainment [39–45], which are three human capital indicators heavily emphasized in the literature and widely characterized as having significant associations with outcomes over life cycle, such as morbidity and mortality, outcomes in labor and marriage markets, and fertility and parenting. Our analysis uses prospective longitudinal data from Brazil, Guatemala, Philippines and South Africa. These countries vary substantially in geography, culture and demographic composition of the population, while sharing similar constraints in socioeconomic development and limited health care services. The preterm birth prevalence varies from 8% in Guatemala and South Africa, 9% in Brazil to 15% in Philippines in 2010 [46]; LBW prevalence ranges from 9% in Brazil, 10% in South Africa, 11% in Guatemala, to 21% in the Philippines during 2009–2013 [47]. The maternal mortality per 100,000 live births ranges from 69 in Brazil, 120 in the Philippines to 140 in Guatemala and South Africa. These countries all suffer from medium to high income inequality, with the Gini index being lowest at 43 for the Philippines and highest at 65 for South Africa [48].

## Methods

### Four cohorts data

We analyzed secondary data from four of the five Consortium of Health-Orientated Research in Transitioning Societies (COHORTS) studies, details of which previously have been published: Brazil, Guatemala, Philippines, and South Africa (Table 1) [49–53]. The fifth site, in India, was not included because prenatal care information was not available. Study cohorts were recruited during pregnancy in Guatemala, Philippines and South Africa or at birth in Brazil, and have been followed prospectively through young adulthood. In this secondary data analysis we only used de-identified data and there were no human subjects. We discussed the analysis with the IRB of University of Pennsylvania which waived the requirement for review.

**Table 1:** Characteristics of four cohort studies and the collection of prenatal care visits data.

No	Cohort name	Location	Baseline survey and initial sample size (N)	Year of follow-up used in analysis and number examined (N)	Initial cohort
**1**	Pelotas 1982 Cohort Studies	Pelotas, Brazil	1982, 5914	2005, 4297	Children born in the city’s maternity hospitals (>99% of all births) during 1982. All social classes included.
**2**	The Institute of Nutrition of Central America and Panama (INCAP) Longitudinal Study	Rural Guatemala	1969–77, 2392	2004, 1571	Intervention trial of a high-energy and protein supplement in women, and children <7 years in 1969 and born during 1969–1977 in 4 villages
**3**	Cebu Longitudinal Health and Nutrition Survey (CLHNS)	Cebu (the Philippines)	1983, 3080	2005, 2032	Pregnant women living in 33 randomly selected neighborhoods; 75% urban. All social classes included.
**4**	1990 Birth to Twenty (BT20)	Soweto-Johannesburg (South Africa)	Apr 23-Jun 8 1990, 3273	2009, 2225	Babies born to pregnant women living in a defined urban geographical area. Predominantly poor, black sample.

Note: Each COHORTS data set varies in the way that prenatal care data was collected: 1) Mothers were asked at birth about number of antenatal clinic visits and the pregnancy month initiating the visits in the Pelotas Cohort 1982; 2) The pregnant month at each trimester prenatal exam was recorded by researchers in the Guatemalan sample. No visit if the information is missing; 3) At the 6-7th month pregnancy pregnant women were asked the number of prenatal care visits and the pregnant month of first visit to each of public/private doctor/nurse/midwife and traditional midwife/healer in CLHNS. The final definition would be the total number of visits to all public/private types of care provider and the timing of the first visit to any public/private care provider; 4) Number of prenatal care visits and timing of the first visit was asked three times in the survey: prenatal, 6 months and 3 years old of baby in BT20. However not all individuals have three answers. Data from 6 months survey were used preferentially, and then 3 years old followed by prenatal one.

### Prenatal care variables

The methods used to assess prenatal care varied among the sites (Table 1). We derived three common measures of prenatal care: (1) whether a woman accessed any prenatal care when pregnant with the target child; (2) the total number of prenatal care visits during that pregnancy; and (3) whether the woman had a prenatal care visit in the first trimester of that pregnancy. In the Philippines, we excluded visits to traditional midwives or healers as they only provide massage and herbal medicine [54] (20% of pregnant women visited only traditional midwives by the 6^th^-7^th^ pregnancy month; 36% of pregnant women visited traditional midwives or healers more frequently than public or private ones; and 36% of women went to traditional midwives or healers for the initial visit). For Guatemala, where the sample was recruited in the context of a nutritional supplementation trial and the prenatal care was delivered by the research project team, the number of visits corresponds to the number of study-related assessments and not to measures of routine care, which was not available in the study villages at that time. As there was a maximum of one study-related assessment per trimester, the number of visits is truncated at 3 for this site. In South Africa, information on the first visit was requested in each of the antenatal, 6 months, and 3 year surveys; we used the earliest available postnatal response to minimize the duration of recall periods.

In the Philippines and for some of the South African sample, the number of prenatal care visits was ascertained only during pregnancy and hence information on the total number of prenatal care visits by birth was incomplete. For these individuals we predicted the total number of prenatal care visits by assuming that their prenatal visits followed a common trajectory shared among individuals within the same sample. We regressed the reported number of visits on a polynomial function of gestational age at interview date and the confounders as indicated later in “other variables” section (separately for urban and rural residents for the Philippines), and then predicted the total number of prenatal care visits at the gestational age at birth (with residuals included to capture how individual women differed from the prediction based on observed characteristics due to their individual-specific unobserved characteristics). It is important to note that the schedule of recommended PNC visits calls for more frequent visits in later pregnancy, so a linear regression may underestimate the total number of visits. The prediction models were chosen based on the distribution of the number of visits in each country. For the Philippines, where “no visit” was commonly observed by the time of survey (32%), we fitted a negative binomial model to accommodate the over-dispersed count variable. Most South Africans had at least one visit, so a log-linear regression was used. We checked the validity of the prediction method by plotting the predicted number of visits at birth against the observed number of visits at 6-month interviews for the small group of South Africans who answered the same question in both the antenatal and 6-month-after-birth interviews (and thus for whom we can observe the real number of visits at birth). The plotted graph matched very closely the 45 degree line ({Citation} Fig). We could not do the same test for the Philippines as all information on the number of prenatal visits was collected at 6–7 months of pregnancy. In the analysis below, the predicted number of prenatal care visits at birth was used for these two countries whenever the “number of visits” is mentioned.

### Prenatal care indices

We generated a binary variable indicating whether a woman had more than the site-specific median number of prenatal care visits [15,55]. We created an index of prenatal care utilization by summing values across the three binary measures, i.e., (1) whether a woman accessed any prenatal care when pregnant with the target child; (2) whether a woman had more than the site-specific median number of prenatal care visits; and (3) whether the woman had a prenatal care visit in the first trimester of that pregnancy. This index has four integer values: 0, 1, 2 and 3. We also created an index of overall access using the World Health Organization (WHO) recommended minimum of four prenatal visits to define adequacy. This index could not be computed for the Guatemalan site as the number of prenatal care visits was truncated at 3 according to the research protocol (any additional visits are not captured in the data we have). Finally, we constructed an index based on the Graduated Prenatal Care Utilization Index (GINDEX) [56]. This index synthesizes information on the timing and frequency of prenatal care and takes into consideration the gestational age at delivery. The index has five values: intensive, adequate, intermediate, inadequate, and no care (please see S3 File for the detailed definition of INDEX3). We note that no index is perfect: the first does not use a uniform reference point; the second cannot be calculated for the Guatemalan sample; and the third is defined according to United States’ standards of adequacy that may not be applicable to LMICs.

### Outcome variables

Birth weight was evaluated by birth attendants using pediatric scales that were calibrated weekly by the research team in Brazil. It was measured by the research teams in Guatemala. In the Philippines, birth weight was measured by birth attendants provided with hanging scales for home births or was obtained from hospital records for hospital births. In South Africa, birth weight was obtained from birth records measured by birth attendants [30]. Height or length measurements at 24 months were taken in all four sites and were converted to z scores by comparing them to the 2006 WHO growth standards [32]. The highest grade of school successfully completed was collected in all four cohorts, and it was censored for Brazil and South Africa as some individuals (41.7% for Brazil and 23.4% for South Africa) were still in school at the time of the survey data used in this paper.

### Other variables

In line with previous studies [57–61], controls for confounders in this paper include: maternal schooling, age, height, race (for Brazil and South Africa), marital status, household composition, assets, father's occupational class, and urban/rural residence (for the Philippines). These confounders were all measured at or close to the time of birth. In the pooled regressions, site was included as a set of dummy variables. Gestational age was estimated from date of mother’s last menstrual period (LMP) in Cebu, Brazil and South Africa, or LMP aided by surveillance in Guatemala. In particular in Cebu, for infants weighing <2500 gms and those whose mothers had pregnancy complications, gestational age was estimated by the Ballard method [62].

### Inclusion and exclusion from analysis

We included 7,203 individuals with information on all three outcome variables. The share of the original sample utilized ranged from 31.7% in Guatemala to 62.8% in the Philippines (the low share for the Guatemalan sample is due to the study design, such that births after 1975 were not followed at 24 months due to study closeout). Among these 7203 individuals, 470 observations (18 from Brazil, 56 from the Philippines and 396 from South Africa) are missing information on the number of prenatal care visits. For the Philippines and South Africa, these are observations still missing after the extrapolation described in the above section. Specifically, no information on the number of prenatal visits was available. Among these 7203 individuals, 2674 (2352 from Brazil, 27 from the Philippines and 295 from South Africa) are missing information on the timing of the initial visit, 790 (711 from Brazil, 44 from Guatemala, 17 from the Philippines and 18 from South Africa) are missing information on gestational age, and 906 are missing information on one or more of the confounding variables included. The reason that there were so many observations in South Africa with missing information on prenatal care is because a major hospital strike lasting 6 weeks occurred during the period of recruitment. We do not think this strike event affects the randomness of the missing observations in that the strike was throughout the whole city and the occurrence of the strike was unrelated to pregnancy due dates. In the Brazilian sample, a flaw in the flow of questions led to the question on the timing of the initial visit being skipped frequently.

### Statistical methods

To preserve sample size, we estimated imputation models with the assumption that information on the missing variables was “missing at random”. We generated two different imputation models. First, we imputed gestational age, timing and frequency of prenatal care visits and indices jointly [63], as well as all the other control variables. Second, we imputed only the control variables. As both methods gave very similar results, we present the results of the first method (results with the second method are in S2 Table). We also implemented the analysis on the sample of individuals with complete information on all of the three outcome variables (S3 Table).

We used ordinary least squares (OLS) regressions, with multivariate controls for confounding variables. We treated the first 2 indices as continuous variables with higher values implying earlier and more frequent service, while the third index was treated as a categorical variable because intensive prenatal care may imply an identified high-risk pregnancy that may introduce nonlinearities. We estimated two types of models; non-mediation (unconditional) models that do not control for earlier outcomes in estimating later outcomes; and mediation (or conditional) models in which earlier outcomes are included as right-side controls (i.e., BW is included in models for HAZ at 24 months and schooling attainment, and HAZ at 24 months is included in the model for schooling attainment). Mediation models assume that the associations of prenatal care with earlier and later child human capital outcomes are sequential and unidirectional and estimate the additional association of prenatal care with each later outcome, over and above any associations with earlier outcomes. Estimates were made for the pooled sample and for country-specific samples. Estimates were not differentiated by gender because a test of heterogeneity of coefficients by gender failed to reject the null hypothesis. All estimations were conducted using Stata 13.

## Results

### Prenatal care utilization and descriptions of key variables

Utilization of any prenatal care was very high in South Africa (96.7%) and Brazil (96.1%), and high in Guatemala (74.0%) and the Philippines (68.9%) (Table 2). The Guatemalan women had at most three visits during pregnancy recorded due to the study design, but the majority of the women in the other three sites had at least four visits. A large proportion of women in Brazil (55.7%) and Guatemala (44.6%) had their initial visit in the first trimester, while fewer women in the Philippines (13.9%) and South Africa (19.8%) did so. The summary distribution across the indices of prenatal care is provided in Table 3. The first two indices are highly correlated (r = 0.93), and there is substantial overlap between these indices and the third index (S1 Table). Our presentation therefore focuses on the first index.

**Table 2:** Selected prenatal care characteristics of mothers of participants in four birth cohorts.

	Brazil (n = 3634)	Guatemala (n = 489)	Philippines (n = 1935)	South Africa (n = 1145)
**1. Ever had prenatal care visit**				
Missing	17 (0.47%)	0	18 (0.93%)	293 (25.6%)
Yes	3476 (96.1%)	362 (74.0%)	1321 (68.9%)	824 (96.7%)
**2. Number of prenatal care visits**				
Missing	18 (0.50%)	0	56 (2.89%)	396 (34.6%)
0	141 (3.90%)	127 (26.0%)	596 (31.7%)	28 (3.74%)
1	64 (1.77%)	70 (14.3%)	10 (0.53%)	7 (0.93%)
2	103 (2.85%)	99 (20.3%)	249 (13.3%)	28 (3.74%)
3	184 (5.09%)	193 (39.5%)	274 (14.6%)	56 (7.48%)
4	240 (6.64%)		225 (12.0%)	104 (13.9%)
5	360 (9.96%)		138 (7.34%)	86 (11.5%)
6	457 (12.6%)		129 (6.87%)	107 (14.3%)
7	499 (13.8%)		78 (4.15%)	75 (10.0%)
8	561 (15.5%)		62 (3.30%)	92 (12.3%)
9	460 (12.7%)		30 (1.60%)	35 (4.7%)
>9	547 (15.1%)		88 (4.68%)	131 (17.5%)
**3. Visit in the first trimester**				
Missing	2352 (64.7%)	0	27 (1.40%)	295 (25.8%)
Yes	714 (55.7%)	218 (44.6%)	265 (13.9%)	168 (19.8%)

Note: The numbers are before imputation. Data are frequencies and percentages within each cohort. For missing value cases, the percentage represents the proportion of the cohort sample, and for nonmissing value cases, the percentage represents the proportion of the nonmissing cases of each cohort. For Filipinos and some South Africans, **Number of prenatal care visits** is predicted from the number reported at 6–7 months of pregnancy and rounded up to the next integer.

**Table 3:** Distribution of three prenatal care utilization indices among mothers of participants in 4 birth cohorts.

	Brazil (n = 3634)	Guatemala (n = 489)	Philippines (n = 1935)	South Africa (n = 1145)
**INDEX1**				
Missing #	2353 (64.7%)	0	65 (3.36%)	403 (35.2%)
0	141 (11.0%)	127 (26.0%)	596 (31.9%)	28 (3.77%)
1	383 (29.9%)	67 (13.7%)	487 (26.0%)	251 (33.8%)
2	276 (21.6%)	80 (16.4%)	569 (30.4%)	365 (49.2%)
3	481 (37.6%)	215 (44.0%)	218 (11.7%)	98 (13.2%)
**INDEX2**				
Missing #	2353 (64.7%)		65 (3.36%)	403 (35.2%)
0	141 (11.0%)		596 (31.9%)	28 (3.77%)
1	107 (8.35%)		487 (26.0%)	79 (10.7%)
2	331 (25.8%)		569 (30.4%)	520 (70.1%)
3	702 (54.8%)		218 (11.7%)	115 (15.5%)
**INDEX3**				
Missing #	2558 (70.4%)	26 (5.32%)	65 (3.36%)	408 (35.6%)
No visits	141 (13.1%)	127 (27.4%)	596 (31.9%)	28 (3.80%)
Inadequate care	401 (37.3%)	334 (72.1%)	1006 (53.8%)	383 (52.0%)
Intermediate care	467 (43.4%)	2 (0.43%)	230 (12.3%)	272 (36.9%)
Adequate care	65 (6.04%)	0	29 (1.55%)	26 (3.53%)
Intensive care	2 (0.19%)	0	9 (0.48%)	28 (3.80%)

Note: Data are frequencies and percentages within each cohort. For missing value cases, the percentage represents the proportion of the cohort sample, and for nonmissing value cases, the percentage represents the proportion of the nonmissing cases of each cohort. INDEX1 is the sum of three binary prenatal care variables: ever had prenatal care visits, number of prenatal care visits higher than local medium level and visit in the first trimester. INDEX2 replaces the number of prenatal care visits with a binary variable indicating number of visits greater or equal to the World Health Organization recommended level of 4 visits. INDEX3 is constructed based on the Revised GINDEX in [56]. The total numbers of observations with nonmissing INDEX3 are smaller than the ones with nonmissing INDEX1 because INDEX3 also takes into account gestational age which is missing for 236 children. Analysis of variance shows three indices are all significantly different across sample sites (p<0.005 for all three indices).

There was substantial variability across the four samples with respect to study outcome and control variables (Table 4). Mean BW ranged from 2.97 kg (Guatemala, girls) to 3.24 kg (Brazil, boys), while the prevalence of preterm delivery ranged from 6.0% (Brazil, girls) to 16% (Philippines, boys). Mean HAZ at 24 months was -1.55 and -1.37 for boys and girls respectively, with Guatemalan boys having the lowest and Brazilian girls the highest means. The mean attained schooling for offspring was 9.1 and 9.7 grades for boys and girls respectively, both considerably higher than the means of about 6.6 grades for their mothers. South African girls had the highest mean schooling attainment (11.3 grades), while Guatemalan girls had the lowest (4.7 grades).

**Table 4:** Selected characteristics of participants in 4 birth cohorts, by site and gender.

	Brazil		Guatemala		Philippine		South Africa		Pooled		Difference P	
	Male	Female	Male	Female	Male	Female	Male	Female	Male	Female	Male	Female
**Outcomes**												
Birth weight (kg)	3.24	3.13	3.09	2.97	3.01	2.97	3.12	3.02	3.15	3.05	0.00	0.00
	(0.57)	(0.53)	(0.51)	(0.47)	(0.44)	(0.43)	(0.52)	(0.50)	(0.54)	(0.51)		
Low birth weight (%)*	8.04	10.3	8.91	10.6	10.7	12.4	9.33	12.1	9.03	11.3	0.02	0.04
Height for z score at 2y	-0.78	-0.7	-3.2	-3.01	-2.61	-2.52	-1.29	-1.05	-1.55	-1.37	0.00	0.00
	(1.28)	(1.28)	(1.20)	(1.11)	(1.17)	(1.12)	(1.22)	(1.07)	(1.54)	(1.48)		
Stunted at 2y (%)*	16.3	11.4	84	82.5	69.3	66	24.4	16.6	37.3	32.3	0.00	0.00
Highest grade obtained	8.9	9.61	5.49	4.67	9.49	10.5	10.8	11.3	9.11	9.74	0.00	0.00
	(3.17)	(3.29)	(3.55)	(3.40)	(3.13)	(2.67)	(1.59)	(1.28)	(3.25)	(3.31)		
**Controls**												
Gestational age (weeks)	39.2	39.5	39.1	39.5	38.6	38.9	38.2	38.1	38.8	39	0.00	0.00
	(1.92)	(1.86)	(3.02)	(3.00)	(2.18)	(2.13)	(1.89)	(1.94)	(2.13)	(2.14)		
Preterm (binary)*	6.53	6.03	14.5	11	16	14.5	11.7	12.7	11	10.5	0.00	0.00
Maternal age (y)	25.7	26.3	26.8	27	26.4	26.1	26	25.9	26.1	26.1	0.00	0.00
	(6.00)	(6.35)	(7.28)	(7.36)	(6.10)	(5.92)	(6.07)	(6.09)	(6.21)	(6.26)		
Parity	2.09	2.18	3.03	3	2.7	2.67	2.15	2.09	2.35	2.32	0.00	0.00
	(1.10)	(1.11)	(1.19)	(1.19)	(1.17)	(1.18)	(1.09)	(1.06)	(1.18)	(1.16)		
Only child (%)	40.2	38.3	18.1	18.4	21.9	22.7	35.7	37.5	32.5	32.6	0.00	0.00
Mother's schooling (y)	6.39	6.29	1.4	1.29	7.14	7.07	9.54	9.57	6.7	6.83	0.00	0.00
	(4.11)	(4.31)	(1.64)	(1.57)	(3.37)	(3.24)	(3.00)	(3.01)	(4.15)	(4.21)		
Wealth quintile	2.77	2.77	2.97	2.96	2.84	2.85	2.98	2.96	2.86	2.9	0.00	0.00
	(1.29)	(1.36)	(1.34)	(1.40)	(1.38)	(1.40)	(1.34)	(1.31)	(1.33)	(1.34)		
Maternal height (cm)	156	156	149	149	151	150	159	158	155	155	0.00	0.00
	(6.18)	(6.03)	(5.28)	(5.26)	(5.00)	(5.01)	(6.40)	(6.38)	(6.91)	(6.72)		
Mother is married (%)	91.9	91.7	91	92.8	97.8	97.2	44.3	42.8	82.3	80.6	0.00	0.00
Children dependence	0.66	0.65	1.1	1.13	0.92	0.9	0.5	0.5	0.74	0.72	0.00	0.00
Ratio	(0.35)	(0.35)	(0.61)	(0.58)	(0.50)	(0.50)	(0.33)	(0.33)	(0.46)	(0.45)		
Crowding index	2.94	3.11	4.38	4.31	3.12	3.19	3.27	3.24	3.21	3.21	0.00	0.00
	(1.37)	(1.61)	(2.47)	(2.32)	(1.79)	(1.89)	(1.66)	(1.61)	(1.74)	(1.74)		
Father's occupation	2.19	2.08	2.36	2.33	2.61	2.51	1.91	1.87	2.24	2.19	0.00	0.00
Class	(1.42)	(1.39)	(0.87)	(0.93)	(1.07)	(1.06)	(1.31)	(1.27)	(1.29)	(1.29)		
Toilet type (%)												
*No toilet at home*	0.69	0.37	86.8	88.7	36.3	37.4	0	0	15.8	16.3	0.00	0.00
*Some excreta removal*	21.1	20.2	4.72	5.41	58.3	58	0.65	0.62	28.1	26.2	0.00	0.00
*Flush toilet*	78.3	79.4	8.49	5.86	5.46	4.52	99.4	99.4	56	57.5	0.00	0.00
Water access (%)												
*Worst access to safe water*	5.02	4.33	16.5	23	15.4	18.6	0	0	8.02	8.82	0.00	0.00
*Moderate access to safe water*	18.7	19.3	79.7	69.8	73.2	70.5	14.4	14.1	37.3	35.8	0.00	0.00
*Best access to safe water*	76.3	76.3	3.77	7.21	11.4	10.9	85.7	85.9	54.7	55.4	0.00	0.00

Note: Statistics are mean or %, with standard deviations in parentheses.

* are for the variables not included in analysis as outcome or control variables. Mother’s schooling is years of completed education. Crowding index denotes the ratio of the number of people over the rooms of the house. Occupational class represents father/mother’s occupation in six rank ordered categories, with unemployed as 0, lowest occupational class as 1, moving up to 5 for the highest class such as professional, technical and commercial. Wealth quintile is the household asset score grouped in quintiles for each country site, running from 1 (poorest) to 5 (wealthiest). The definition of asset is also site specific.

Although these four countries vary substantially in various dimensions, the variation in prenatal care utilization indices is mainly within-site (92.4% of variation in index 1, 80.4% in index 2 and 84.0% in index 3 are within-site). This is not surprising given that index 1 is defined against site-specific median values and the high correlations between index 1 and the other two indices. Regressions of the prenatal care indices on SES show that child’s birth order, family’s social class, household wealth, mother’s schooling and marital status, and prenatal care facility accessibility all are associated with prenatal care utilization (S6 Table). Especially in the Philippines, social class and wealth are significant predictors for prenatal care use (this point is also well documented in [58]). A shift from the first to second quintile of wealth is associated with a 0.16 increase in the prenatal care utilization index. Moreover, more-schooled mothers also utilize prenatal care more and earlier in Brazil and the Philippines, while unmarried mothers are less likely to obtain early and frequent prenatal care in Brazil and Guatemala. A binary variable describing accessibility to prenatal-care providers by travel time or distance to care facility is also a strong predictor for prenatal care use in all sites except in Guatemala.

Besides these common factors, each site has specific factors that lead to within-site variation in prenatal care. For example, needs-based prenatal care use is highlighted in rural Guatemala where women of pregnancies with complications are more likely to seek biomedical prenatal care [64]. This point is well-documented in our data by the fact that morbidities during pregnancies are important predictors for prenatal care utilization in Guatemala, although accessing prenatal care was study-driven. The positive and decreasing gradient from the first-trimester morbidity to the third one (although not significantly different from each other) suggests that prenatal-care utilization in Guatemala (see Column 3 in S6 Table) is mainly need-based, while SES and accessibility to health care facilities don’t have much predictive power (F-test statistic for all control variables without morbidities is 0.917, p = 0.569).

### Main results

Prenatal care utilization was not significantly associated with BW in the pooled sample (Table 5). In the non-mediation models, each unit increase in prenatal care utilization was associated with a 0.09 (95% CI 0.04 to 0.15) increase in HAZ at 24 months and with a 0.26 (95% CI 0.17 to 0.35) increase in highest attained schooling grade. These associations were not substantively altered by adjustment in the mediation models. See S7 Table for the complete table of coefficients of all the control variables.

**Table 5:** Associations of maternal prenatal care utilization index with offspring outcomes in four birth cohorts (n = 7203).

	NON-MEDIATION MODELS			MEDIATION MODELS	
	(1)	(2)	(3)	(4)	(5)
Birth weight	0.01 (-0.04–0.07)				
	p = 0.45				
HAZ at 24 months		0.09*(0.04–0.15)		0.09** (0.05–0.12)	
		p = 0.01		p = 0.00	
Highest attained grade			0.26** (0.15–0.36)		0.23** (0.11–0.34)
			p = 0.00		p = 0.01

Notes: Prenatal care utilization index is the sum of three binary prenatal care variables: ever had prenatal care visits, number of prenatal care visits higher than local medium level and visit in the first trimester.

*** p value<0.001,

** p value<0.01,

* p value<0.05.

All the models are adjusted for controls including child’s gender, birth order, whether being the only child at birth, maternal schooling, age, height, race, marital status, household composition, wealth, and occupational class as well as country fixed effects. The mediation model (4) also controls for *birth weight* and mediation model (5) controls for *birth weight* and *HAZ at 24 mo*. Data were analyzed using linear regressions with multiple imputations (20 times) of missing control variables, gestational age, prenatal care variables and prenatal care utilization jointly, with variances clustered at site levels. 95% confidence intervals are reported in parentheses.

### Site heterogeneity

**Fig 1:**
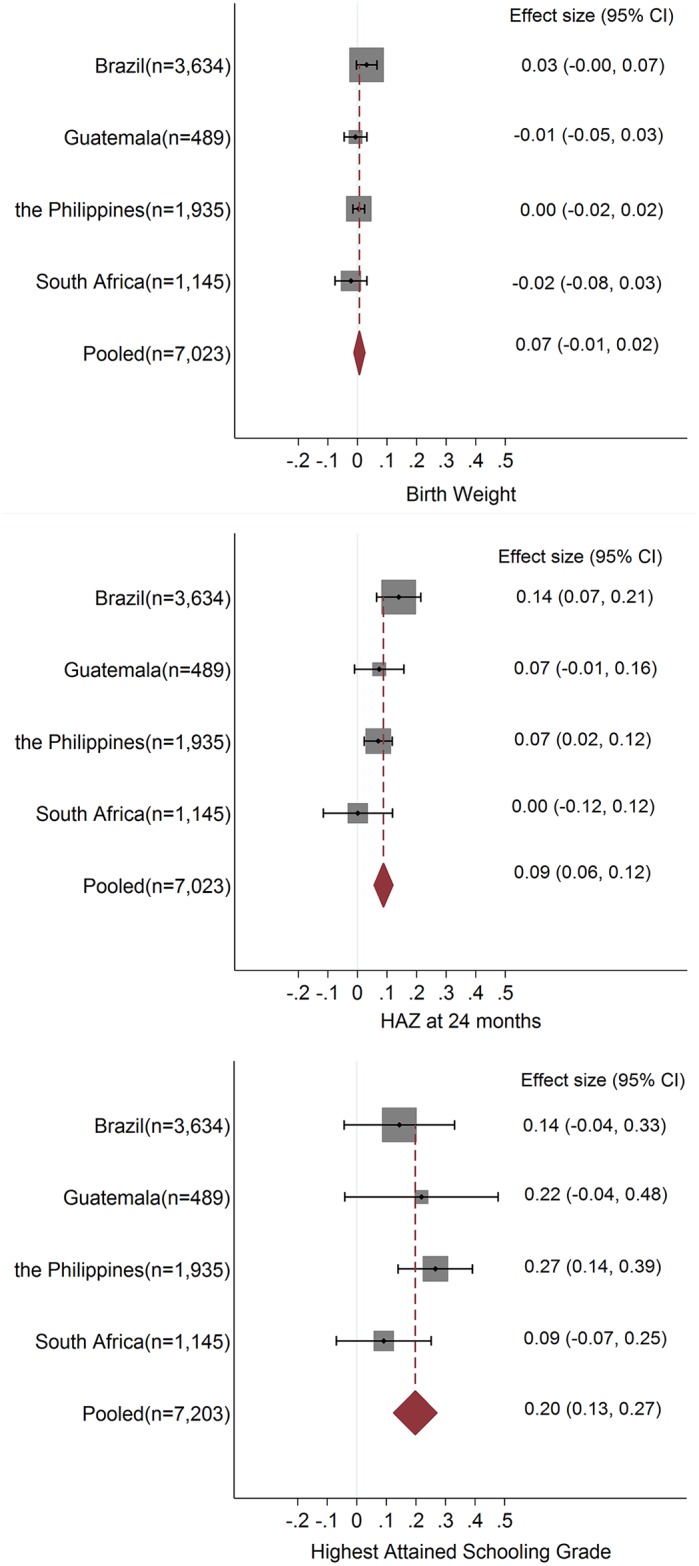
Magnitude of associations and CIs (coefficients) of INDEX1 in non-mediation model. The magnitudes of associations and CIs (coefficients) of INDEX1 with (a) birth weight (b) HAZ at 24m (c) highest attained schooling in non-mediation model. INDEX1 is the sum of three binary prenatal care variables: ever had prenatal care visits, number of prenatal care visits higher than local medium level and visit in the first trimester. The non-mediation (or unconditional) model did not control for earlier outcomes in estimates for later outcomes. The area of each square is proportional to the study's weight in the meta-analysis. The dashed vertical line is the overall meta-analyzed measure of association. The diamond is the meta-analyzed measure of association, the lateral points of which indicate confidence intervals for this estimate.

The association between prenatal care utilization and BW was not significant for individual sites (Fig 1(a)). Although an insignificant aggregate association may be the result of a combination of opposite biases, non-significant individual associations indicate that the true effects of prenatal care utilization on BW are not likely to be significantly different from zero. For example, if the association for the Philippines is upward-biased due to positive selection, the true effect (if positive) would be even lower. Since the estimated association is non-significantly different from zero, it is not very likely that the true effect is significantly positive. For the association between prenatal care utilization and HAZ at 24 months in the non-mediation model, Brazil had the strongest association (+0.14, 95% CI 0.06 to 0.21), followed by the Philippines (+0.07, 95% CI 0.02 to 0.12), while the associations in Guatemala and South Africa were not significant (Fig 1(b)). A similar pattern was observed in the mediation model (S2 Fig).

There was significant heterogeneity across sites for the associations between prenatal care utilization and highest attained schooling grade, in both non-mediation and mediation models. Only for the Philippines sample was there a significant association (+0.27, 95% CI 0.14 to 0.39, for non-mediation model and +0.27, 95% CI 0.15 to 0.40, for mediation model) (S1(c) and S2(b) Figs respectively).

### Robustness to alternative measures of prenatal care utilization

We re-estimated the models using the second index of prenatal care utilization (excluding the Guatemala sample for which it cannot be computed). All the associations remained qualitatively similar, though with greater imprecision for HAZ at 24 months (S4 Table).

We also estimated the model using the GINDEX, with imputation of all missing prenatal care variables, gestational age and GINDEX and other control variables. The GINDEX was significantly associated with all three outcomes in both non-mediation and mediation models (S5 Table).

Finally we considered each individual component of prenatal care separately (Table 6). The individual prenatal care variables were each significantly associated with highest schooling grade attained in non-mediation models, with *ever-visit* having a stronger association (+0.66, 95% CI 0.08 to 1.23) than either *visit in the first trimester* (+0.30, 95% CI 0.02 to 0.58) or *the number of visits* (+0.08, 95% CI 0.02 to 0.15). The number of prenatal visits was positively associated with BW; none of the three components was significantly associated with HAZ at 24 months.

**Table 6:** Pooled analysis of birth and later outcomes with individual prenatal care visit indicators (N = 7203).

	NON-MEDIATION MODELS			MEDIATION MODELS	
	(1)	(2)	(3)	(4)	(5)
**Ever visit (yes = 1)**					
Birth weight	0.04(-0.11–0.19)				
	p = 0.50				
HAZ at 24 months		0.24(-0.13–0.61)		0.21	
		p = 0.11		p = 0.07	
Highest attained grade			0.66*(0.08–1.23)		0.57*(0.13–1.02)
			p = 0.02		p = 0.01
**Number of visits**					
Birth weight	0.01*(0.00–0.02)				
	p = 0.05				
HAZ at 24 months		0.03(-0.01–0.07)		0.02(-0.01–0.06)	
		p = 0.08		p = 0.11	
Highest attained grade			0.09*(0.04–0.15)		0.08*(0.04–0.12)
			p = 0.01		p = 0.01
**Visit in 1st trimester (yes = 1)**					
Birth weight	-0.03(-0.14–0.08)				
	p = 0.46				
HAZ at 24 months		0.08(-0.02–0.18)		0.1(-0.01–0.20)	
		p = 0.13		p = 0.08	
Highest attained grade			0.30*(0.02–0.58)		0.27*(-0.01–0.55)
			p = 0.03		p = 0.04

Note:

*** p value < 0.001,

** p value < 0.01,

* p value < 0.05.

Data were analyzed using linear regressions with multiple imputations (20 times) of missing prenatal care visit variables, gestational age as well as other control variables. The models adjusted for all the controls including maternal schooling, age, height, race, marital status, household composition, wealth, and occupational class. The mediation model (4) also controls for birth weight and the mediation model (5) controls for birth weight and HAZ at 24 mo. 95% confidence intervals, clustered at site level, are reported in parentheses.

### Possible mechanism

We examined the associations between prenatal-care utilization and breastfeeding incidence and duration and, but did not find significant associations for the pooled sample. In Brazil, prenatal care utilization was positively associated with initiation of breastfeeding, but not the duration of breastfeeding, and in the Philippines women with higher prenatal-care utilization index had shorter duration of breastfeeding (S8 Table). This latter finding reinforces the positive selection into prenatal care utilization in the Philippines as the more educated and wealthier Philippine women were less likely to breast feed at the survey time.

## Discussion

We pooled data from birth cohort studies from four LMICs, to examine associations between prenatal care utilization and three important human capital outcomes–BW, HAZ at 24 months and highest schooling grade attained. Prenatal care utilization was not associated with BW, but had significant associations with HAZ at 24 months and highest school grade attained. The association with highest school grade attained was partly mediated through HAZ at 24 months.

We did not find significant associations between prenatal care utilization and BW, either in the pooled sample, or in any individual country. If the estimated associations were reflective of causal effects they were not supportive of the contention that prenatal care can improve this particular important birth outcome. The empirical evidence regarding the association between prenatal care and birth outcomes has been equivocal [10,11,14], leading to the value of current prenatal care being questioned. For example, a counterfactual analysis suggests that the increase in preterm births in the United States is more likely due to changes in obstetric practice, rather than to changes in the demographics of childbearing [65]. A review of trials assessing the effectiveness of different models of prenatal care found that a lower number of prenatal visits from the standard model could be introduced into clinical practice without risk to mother or baby [19]. However, the content of prenatal care varies considerably, especially during the time period when our data were collected. Recent literature has examined the association of specific components of prenatal care on birth outcomes, such as maternal nutrition intervention [66,67], providing psychosocial interventions and counselling [68]. Those aspects of prenatal care were not captured in our data. Clearly, more research should be done on the content and quality of prenatal care, especially in LMICs, which have limited resources for competing uses.

On the other hand, earlier initiation and more frequent utilization of prenatal care predicted child HAZ at 24 months and school attainment. If these predictions are related to causal effects, it is important in evaluating prenatal care to consider mid- and longer-term effects as a focus on birth weight alone would miss important implications of more extensive prenatal care. Most of these estimates were qualitatively similar across the four countries, although with some heterogeneity in magnitudes, which suggested some qualified external validity though still the importance of country-specific contexts. Our results were qualitatively similar across our indices of prenatal care utilization, which is not surprising considering their high degree of interchangeability.

Our study has limitations. First, the methods for recording prenatal care utilization were heterogeneous among the four COHORTS data sets. For example, for the Filipinos and some of the South Africans, the information on the number of prenatal care visits was not complete due to the antenatal timing of the survey; the use of synthesized indices may have attenuated any bias. For the Guatemalan sample, prenatal care utilization did not reflect routine care in the community but study-specific assessments. Importantly, we lack measures of quality of the prenatal care provided. Second, we were limited in our choice of outcome variables by the secondary nature of the data, although the three outcome variables used in this paper were three widely-studied human capital indicators that have strong implications for future outcomes. Moreover, among the three outcome variables studied, birth weight was likely measured with the most error, and hence the lack of association with prenatal care utilization may reflect measurement error.

Third, we controlled for a standard set of observed control variables, but unobserved factors may have introduced bias and hence limit causal inference. For example, mother’s preferences for health and education, usually difficult to measure, may be related both to use of prenatal care and to the child’s health and schooling outcomes. As the data were not collected based on a uniform module across the four sites, we could not find a valid instrumental variable that is commonly available across sites. Though the binary index of accessibility to health-care facilities can be a candidate for an instrumental variable, it was not defined uniformly across countries. And in South Africa, it was defined based on the observed prenatal visit. More importantly, it may not satisfy exclusive restrictions as travel time to health-care facilities used to define the accessibility as in some sites can affect birth outcomes directly and not only through prenatal-care use (for example, living closer to the community center usually implies less travel time for prenatal care, but also implies better access to other public facilities such as food markets and educational facilities). For this reason, all the estimates in this paper are associations rather than causal effects. The associations will be biased downward from the true causal effects if prenatal care utilization is need-based and upward if missing variables positively contribute to prenatal care use and also improve children’s outcomes. However, as the factors that contribute to selectivity of prenatal care use are different across sites, it is difficult to predict the bias direction of the aggregate estimate. This concern is mitigated by the observation that the estimates did not change much from the non-mediation model to the mediation model. If there existed an unobserved factor that was correlated with both prenatal-care utilization and outcome variables, the effect would be partly captured by the intermediate variables in the mediation model, i.e., BW in the model for HAZ-at-24 months, and BW and HAZ-at-24 months in the model for highest attained schooling grade. That is to say, one would expect the coefficients on prenatal care utilization index be smaller in the mediation model compared to the non-mediation model. However, the very similar results of these two models imply a very limited role of these unobserved factors.

Another limitation is that due to data constraints, we were not able to identify the contents of prenatal care that may improve later outcomes. What constitutes good prenatal care is an important question for policy makers, especially if the benefit is not reflected in the measured birth outcomes, but later in life as in our findings. We did not find significant associations between prenatal care utilization and breastfeeding incidence and duration. Therefore, it is unlikely that prenatal care improved later outcomes through promoting breastfeeding for this sample. It is worth noting that a paper using the Philippines’ data alone finds visits to public prenatal care providers to be associated with positive behavioral changes including taking vitamin pills and receiving antitetanus shots [69]. However, studying the content of prenatal care is out of scope of this paper due to the limited availability of variables commonly shared across countries.

Finally, as with any longitudinal study spanning multiple decades, attrition was significant and was differential in terms of several observed characteristics. However, several studies of the Guatemala data set have found that such attrition in terms of observed characteristics did not affect substantially multivariate estimates [70–72]. But it is not known, once again, how roles of unobserved characteristics in attrition affected the estimates.

Our result on birth weight with Philippines data is not consistent with a previous finding on the positive effect of prenatal care utilization on birth weight using the urban sample from the same data set [22]. Using below-average rainfall shocks during pregnancy to instrument for prenatal care utilization for the urban sample, that paper found a positive and significant impact on birth weight. However, if one believes positive selection is the source of upward bias of OLS estimate, one would expect IV estimates to be smaller than OLS ones, instead of being bigger as was found in that paper.

Our study, nevertheless, had considerable strengths in using long-run prospective cohort data that had both birth and later child and schooling outcomes, as well as multiple indicators of prenatal care visits in four different LMIC country contexts. Therefore, we were able to look beyond birth weight and incorporate longer-term child outcomes that might have been affected by prenatal care. The prospectively-collected prenatal care utilization data reduce the risk of measurement errors due to inaccurate maternal recall. Finally, we considered external validity by comparing outcomes in four very different LMIC contexts, and found significant associations with important post-birth child human capital outcomes—HAZ at 24 months and highest attained schooling grade, both of which are predictive of important outcomes later in the life cycle [36,37,73]. LMICs are particularly important because of higher birth rates, much lower provision and utilization of prenatal care, and the many competing demands on the limited resources that might be used to expand prenatal care in these settings. To our knowledge, very few studies have such multi-country longitudinal analysis. One exception is the work by Woodhouse et al. (2014) [74] that examined the association between prenatal care utilization and BW in eight South American countries. They evaluated prenatal care utilization measured by the number of prenatal care visits only, which were significantly (p < .05) and positively associated with BW and negatively associated with LBW, although with large heterogeneity across countries. Our work differs from this paper by including four LMICs from three continents, and more importantly we study both timing and frequency of prenatal care visits and examines their associations with longer-term outcomes beyond birth.

Our study points to some definite needs for future research, perhaps most importantly, assessing to what extent the associations presented in this study are informative regarding causal effects and with regard to measuring and assessing other features of the quality of prenatal care. It is also important to distinguish whether prenatal care induces health-improving behaviors during pregnancy or health-seeking behaviors for children in later periods that improve child outcomes. Nevertheless, this study contributes importantly to knowledge about associations of prenatal care with child outcomes from birth through schooling in varying developing country contexts.

## Supporting information

S1 Fig Observed vs. predicted number of prenatal care visits at 6 months interview for South Africans. Distribution of the number of prenatal care visits at 6 months interview and the predicted number of visits based on information at pregnancy interview for South Africans. The solid line corresponds to the number of prenatal care visits equaling the predicted value, with points below (above) the line indicating under (over) prediction.
(TIF)

S2 Fig Magnitude of associations and CIs (coefficients) of INDEX1 in mediation model. Magnitude of associations and CIs (coefficients) of INDEX1 on (a) HAZ at 24m (b) highest attained schooling in mediation model. INDEX1 is the sum of three binary prenatal care variables: ever had prenatal care visits, number of prenatal care visits higher than local medium level and visit in the first trimester. Mediation (or conditional) model controlled for earlier outcomes in estimates for later outcomes, i.e., birth weight for HAZ at 24m, birth weight and HAZ at 24m for highest attained schooling. The area of each square is proportional to the study's weight in the meta-analysis. Dashed vertical line is the overall meta-analyzed measure of association. The diamond is the meta-analyzed measure of association, the lateral points of which indicate confidence intervals for this estimate.
(TIF)

S1 File Final minimal dataset. (CSV)

S2 File Description of the variables in the data. (DOCX)

S3 File Definition of INDEX3. (DOCX)

S1 Table Cross-classification of two indicators of prenatal care utilization in four birth cohorts. INDEX1 is the sum of three binary prenatal care variables: ever had prenatal care visits, number of prenatal care visits higher than local medium level and visit in the first trimester. INDEX3 was defined according to the Revised GINDEX as in [50].
(DOCX)

S2 Table Associations of maternal prenatal care utilization index with offspring outcomes in four birth cohorts. The maternal prenatal care utilization index is the sum of three binary prenatal care variables: ever had prenatal care visits, number of prenatal care visits higher than local medium level and visit in the first trimester. *** p value<0.001, ** p value<0.01, * p value<0.05. The models adjusted for controls including maternal schooling, age, height, race, marital status, household composition, wealth, and occupational class. The mediation model (4) also controls for birth weight and the mediation model (5) controls for birth weight and HAZ at 24 mo. Data were analyzed using linear regressions with **multiple imputations (20 times) of missing control variables only**. 95% confidence intervals are reported in parentheses.
(DOCX)

S3 Table Associations of maternal prenatal care utilization index with offspring outcomes in four birth cohorts (maximum sample). The maternal prenatal care utilization index is the sum of three binary prenatal care variables: ever had prenatal care visits, number of prenatal care visits higher than local medium level and visit in the first trimester. *** p value<0.001, ** p value<0.01, * p value<0.05. The models adjusted for controls including maternal schooling, age, height, race, marital status, household composition, wealth, and occupational class. The mediation model (4) also controls for birth weight and mediation model (5) controls for birth weight and HAZ at 24 mo. Data were analyzed using linear regressions with **multiple imputations (20 times) of missing control variables only,** with variances clustered at site level. 95% confidence intervals are reported in parentheses.
(DOCX)

S4 Table Associations of alternative maternal prenatal care utilization index with offspring outcomes in four birth cohorts. The alternative maternal prenatal care utilization index is the sum of three binary prenatal care variables: ever had prenatal care visits, number of prenatal care visits **higher than or equal to** the World Health Organization recommended level– 4 visits—and visit in the first trimester. *** p value<0.001, ** p value<0.01, * p value<0.05. The sample includes all observations except Guatemalans. The models adjusted for controls including maternal schooling, age, height, race, marital status, household composition, wealth, and occupational class. The mediation model (4) also controls for birth weight and mediation model (5) controls for birth weight and HAZ at 24 mo. Data were analyzed using linear regressions with multiple imputations (20 times) of missing control variables, gestational age, prenatal care variables and index jointly with variances clustered at site level. 95% confidence intervals are reported in parentheses.
(DOCX)

S5 Table Pooled analysis of birth and later outcomes with INDEX3. INDEX3 was defined according to the Revised GINDEX in [50]. *** p value<0.001, ** p value<0.01, * p value<0.05. The models adjusted for controls including maternal schooling, age, height, race, marital status, household composition, wealth, and occupational class. The mediation model (4) also controls for birth weight and mediation model (5) controls for birth weight and HAZ at 24 mo. Data were analyzed using linear regressions with multiple imputations (20 times) of missing control variables, gestational age, prenatal care variables and INDEX3 jointly, with variances clustered at site level. 95% confidence intervals are reported in parentheses.
(DOCX)

S6 Table Associations of SES and other household characteristics with prenatal care utilization index in four birth cohorts. The sample consists of all the observations without missing prenatal care utilization index in the original sample. The index is the sum of three binary prenatal care variables: ever had prenatal care visits, number of prenatal care visits higher than local medium level and visit in the first trimester. Mother’s schooling is years of completed education. Crowding index denotes the ratio of the number of people over the rooms of the house. Occupational class represents father/mother’s occupation in six rank ordered categories, with unemployed as 0, lowest occupational class as 1, moving up to 5 for the highest class such as professional, technical and commercial. Wealth quintile is the household asset score grouped in quintiles for each country site, running from 1 (poorest) to 5 (wealthiest). The definition of asset is also site specific. Accessibility index to health care facilities is a binary variable with 1 for good access to health services, and defined site specifically: Brazil defined it based on principal component of a few continuous variables in assessing accessibilities; for Guatemala, it is defined based on the distance from home to supplement center where antenatal care is implemented during the study period; for the Philippines, it is defined based on the average travel time to the closest public or private health facility; and for the South Africa, poor accessibility is defined if the mother reported travelling over 45 minutes to her nearest well-baby clinic or if reported no antenatal care. Morbidity at 1st-3rd trimester for Guatemala are the number of types of morbidity (among three types: respiratory disease, diarrhea, fever) during each trimester self-reported by the pregnant mothers to survey enumerator, in contrast to medical records which are more likely endogenous. *** p value<0.001, ** p value<0.01, * p value<0.05. Data were analyzed using linear regressions with multiple imputations (20 times) of missing control variables, with variances clustered at site level. 95% confidence intervals are reported in parentheses.
(DOCX)

S7 Table Associations of maternal prenatal care utilization index and all the controls with offspring outcomes in four birth cohorts. Prenatal care utilization index is the sum of three binary prenatal care variables: ever had prenatal care visits, number of prenatal care visits higher than local medium level and visit in the first trimester. *** p value<0.001, ** p value<0.01, * p value<0.05. All the models are adjusted for controls including child’s gender, birth order, whether being the only child at birth, maternal schooling, age, height, race, marital status, household composition, wealth, and occupational class as well as country fixed effects. The mediation model (4) also controls for *birth weight* and the mediation model (5) controls for *birth weight* and *HAZ at 24 mo*. Data were analyzed using linear regressions with multiple imputations (20 times) of missing control variables, gestational age, prenatal care variables and prenatal care utilization jointly, with variances clustered at site level. 95% confidence intervals are reported in parentheses.
(DOCX)

S8 Table Associations between prenatal care utilization index and breastfeeding incidence and duration. For Guatemala sample, there is no enough variation in breastfeeding incidence for estimation (only 1 out of 489 observations having no breastfeeding). Data were analyzed using probit model (for breastfeeding incidence) and linear regression (for breastfeeding duration) with multiple imputations (20 times) of missing control variables, gestational age and prenatal care utilization index jointly, with variances clustered at site level. 95% confidence intervals are reported in parentheses.
(DOCX)
