# Supplementary figures and images for: Prenatal care and child growth and schooling in four low- and medium-income countries
Source: PLoS One. 2017 Feb 3;12(2):e0171299. doi: 10.1371/journal.pone.0171299 (PMC5291430; doi:10.1371/journal.pone.0171299)

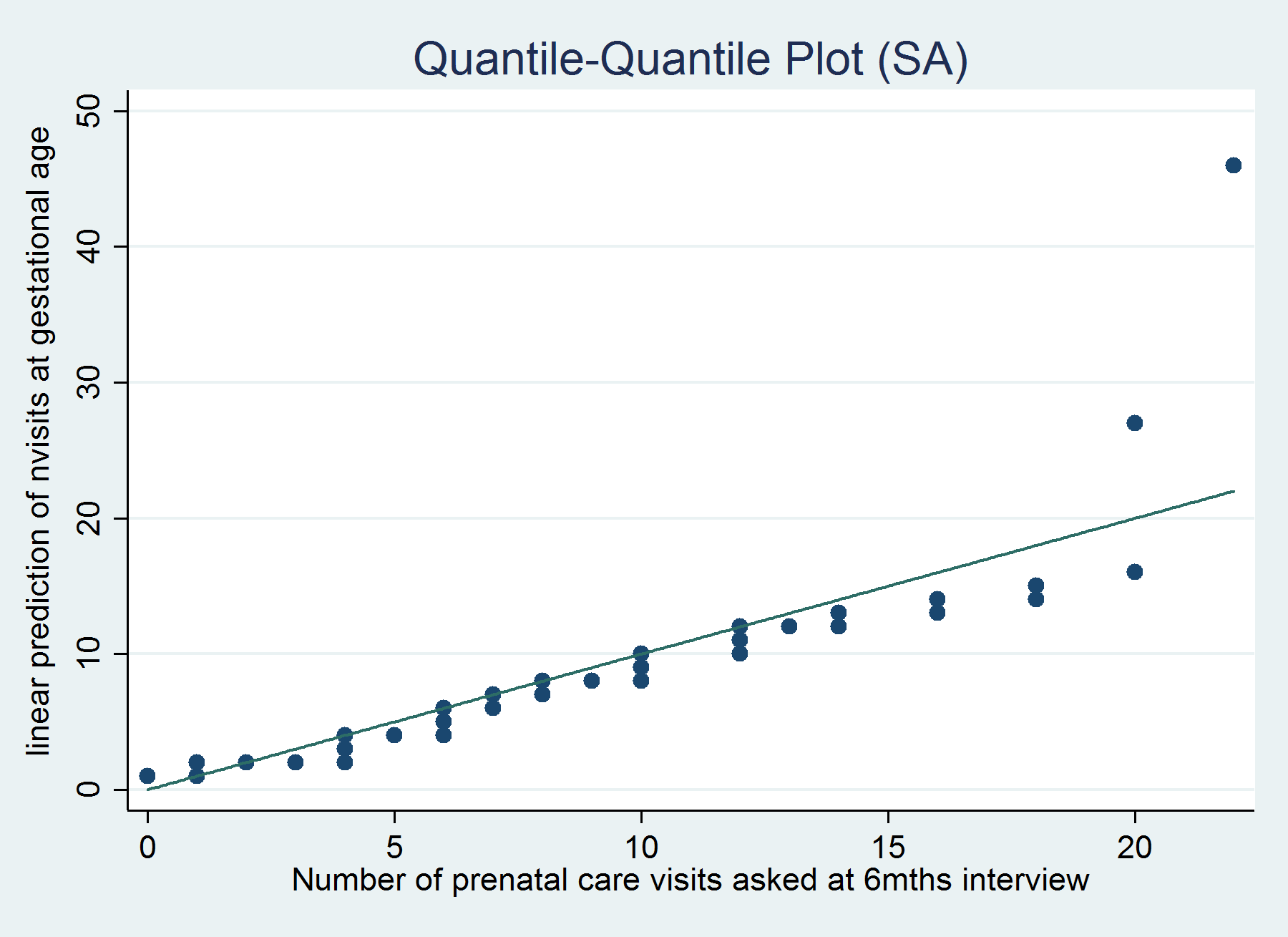

Supplement: S1 Fig — Distribution of the number of prenatal care visits at 6 months interview and the predicted number of visits based on information at pregnancy interview for South Africans. The solid line corresponds to the number of prenatal care visits equaling the predicted value, with points below (above) the line indicating under (over) prediction. (TIF) [file pone.0171299.s001.tif]

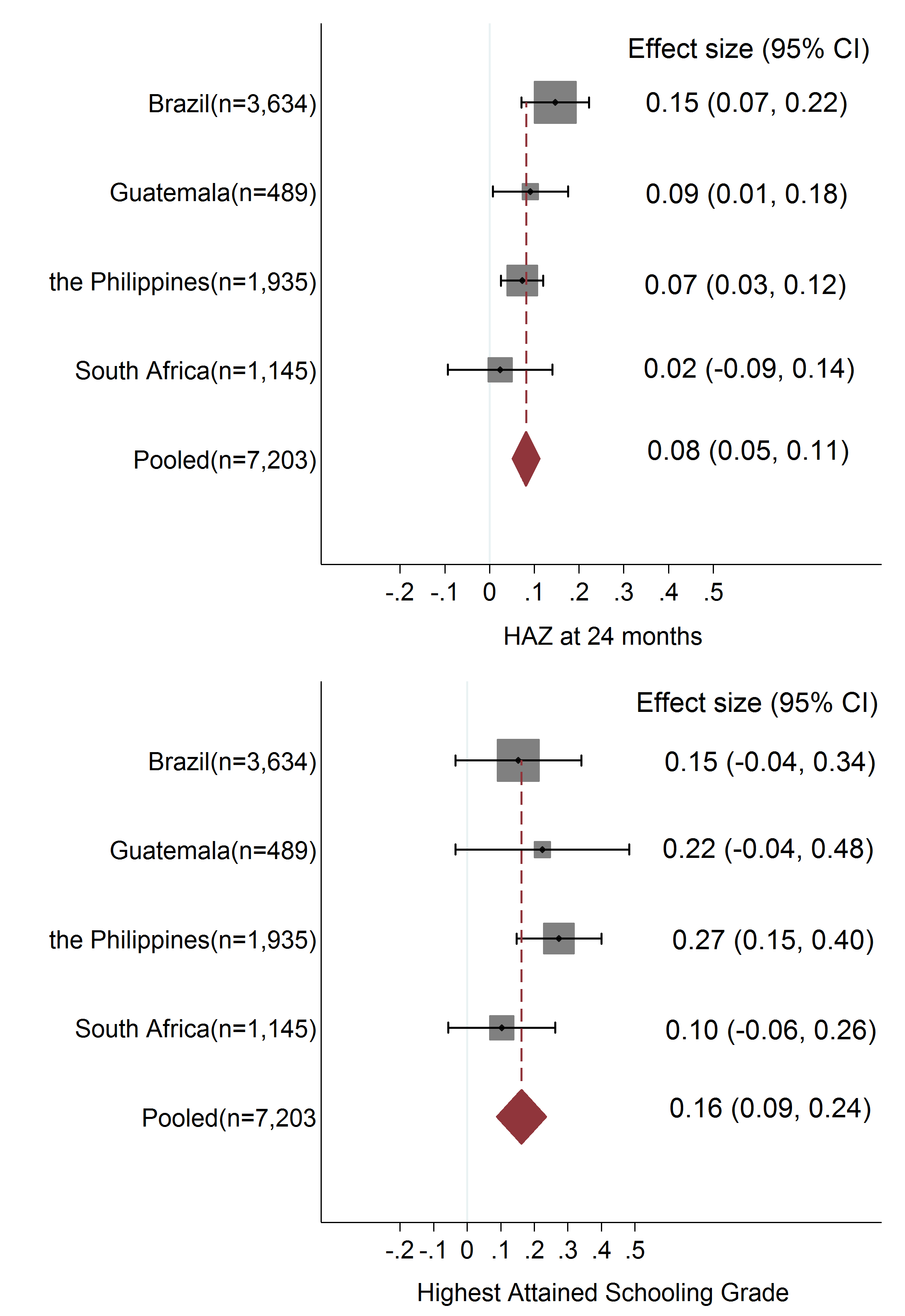

Supplement: S2 Fig — Magnitude of associations and CIs (coefficients) of INDEX1 on (a) HAZ at 24m (b) highest attained schooling in mediation model. INDEX1 is the sum of three binary prenatal care variables: ever had prenatal care visits, number of prenatal care visits higher than local medium level and visit in the first trimester. Mediation (or conditional) model controlled for earlier outcomes in estimates for later outcomes, i.e., birth weight for HAZ at 24m, birth weight and HAZ at 24m for highest attained schooling. The area of each square is proportional to the study's weight in the meta-analysis. Dashed vertical line is the overall meta-analyzed measure of association. The diamond is the meta-analyzed measure of association, the lateral points of which indicate confidence intervals for this estimate. (TIF) [file pone.0171299.s002.tif]
