# Supplementary material for: Prenatal care and child growth and schooling in four low- and medium-income countries
Source: PLoS One. 2017 Feb 3;12(2):e0171299. doi: 10.1371/journal.pone.0171299 (PMC5291430; doi:10.1371/journal.pone.0171299)
Supplement: S2 File — (DOCX) [file pone.0171299.s004.docx]

## Description of the variables in the data

| newid | Unique id to identify each individual |
| --- | --- |
| site | Site code for each country site |
| nvisits | # prenatal care visits at birth for Brazil and Guatemala and part of SA, and predicted # ANC visits at birth for the Philippines and part of SA |
| invisits | =1, if # prenatal care visits predicted at individual gestational age |
| nvisits_public | # prenatal care visits to public providers, including doctors, nurses, midwives and other personals at interview time , for the Philippines |
| nvisits_private | # prenatal care visits to private providers, including doctors, nurses, midwives at interview time, for the Philippines |
| nvisits_traditional | # prenatal care visits to traditional providers, including mananabang and mananambal at interview time, for the Philippines |
| firstvisit | The month in pregnancy of first prenatal care visit |
| ga | Gestational age at birth |
| wt0 | Weight at birth, kg |
| hazwho24 | WHO height z-score at 24 mo |
| urban | Binary, 1 = urban site, 0 = rural site |
| c3hgrade | Highest school grade attained |
| c3toilet (sanitation) | Category variable, 0=no toilet; 1=some excreta removal; 2=flush toilet |
| c3water (water supply) | Category variable, 0= worst access to safe water; 1=moderate access to safe water; 2=best access to safe water |
| c3crowd | Crowding factor, = #people / #rooms |
| c3chdep | Child dependency ratio, = #children under 5/#adults |
| c3soclass (occupational class) | six rank ordered categories, with unemployed as 0; lowest occupational class as 1, moving up to 5 for the highest class (professional, technical, commercial) – each site to provide details for all 6 categories. |
| c3wealthq (assets reported in quintiles by site) | Assets score grouped in quintiles, running from 1 (poorest) to 5 (wealthiest). Assets are defined by each site. |
| c3matage | Maternal age at delivery of the child, years |
| c3mtscho | Maternal education measured in years of completed education |
| c3matht | Maternal height, rounded **cm** |
| c3marst | Maternal marital status. 0=single or not living together 1=married (any definition) or living together. |
| c3sex | 1=male, 2=female |
| c3birtho (birth order of index child) | 1=first born; 2=second born; 3=third born; 4=born fourth or later |
| c3onlych | 0=not an only child at birth; 1=only child at birth |
