# Supplementary material for: Prenatal care and child growth and schooling in four low- and medium-income countries
Source: PLoS One. 2017 Feb 3;12(2):e0171299. doi: 10.1371/journal.pone.0171299 (PMC5291430; doi:10.1371/journal.pone.0171299)
Supplement: S3 File — (DOCX) [file pone.0171299.s005.docx]

INDEX3 is generated according to Graduated Prenatal Care Utilization Index (GINDEX). It has five values: 0, 1, 2, 3, 4, which stands for no care, inadequate care, intermediate care, adequate care and intensive care separately.

Abbreviation: ga -- gestational age

- Intensive care level

INDEX3 = 4 if the first visit is in the **first** trimester AND

(((ga>=18 AND ga<=21) AND # visits>=11)

OR ((ga>=22 AND ga<=25) AND #visits>=13 )

OR ((ga>=26 AND ga<=29) AND # visits>=14)

OR ((ga>=30 AND ga<=31) AND # visits>=15)

OR ((ga>=32 AND ga<=36) AND # visits>=16)

OR ((ga>=37 AND ga<=40) AND # visits>=17)

OR ((ga>=41 AND ga<=42) AND # visits>=18)

OR ((ga>=43 AND ga<=45) AND # visits>=19));

OR if the first visit is in the **second** trimester AND

(((ga>=18 AND ga<=21) AND # visits>=10)

OR ((ga>=22 AND ga<=25) AND # visits>=11)

OR ((ga>=26 AND ga<=31) AND # visits>=12)

OR ((ga>=32 AND ga<=35) AND # visits>=13)

OR ((ga>=36 AND ga<=37) AND # visits>=14)

OR ((ga>=38 AND ga<=40) AND # visits>=15)

OR ((ga>=41 AND ga<=42) AND # visits>=16) OR ((ga>=43 AND ga<=45) AND # visits>=17 ));

OR if the first visit is in the **third** trimester AND

(((ga = 25) AND # visits>=9)

OR ((ga>=26 AND ga<=31) AND # visits>=10 )

OR ((ga>=32 AND ga<=35) AND # visits>=11 )

OR ((ga>=36 AND ga<=37) AND # visits>=12 )

OR ((ga>=38 AND ga<=40) AND # visits>=13 )

OR ((ga>=41 AND ga<=42) AND # visits>=14 ) OR ((ga>=43 AND ga<=45) AND # visits>=15 ));

- Adequate care level

INDEX3 = 3 if the first visit is in the **first** trimester AND

(((ga>=18 AND ga<=21) AND (# visits>=3 AND # visits<=10))

OR ((ga>=22 AND ga<=25) AND (# visits>=4 AND # visits<=12))

OR ((ga>=26 AND ga<=29) AND (# visits>=5 AND # visits<=13))

OR ((ga>=30 AND ga<=31) AND (# visits>=6 AND # visits<=14))

OR ((ga>=32 AND ga<=33) AND (# visits>=7 AND # visits<=15))

OR ((ga>=34 AND ga<=35) AND (# visits>=8 AND # visits<=15))

OR ((ga =36) AND (# visits>=9 AND # visits<=15))

OR ((ga = 37) AND (# visits>=10 AND # visits<=16))

OR ((ga = 38) AND (# visits>=11 AND # visits<=16))

OR ((ga = 39) AND (# visits>=12 AND # visits<=16))

OR ((ga = 40) AND (# visits>=13 AND # visits<=16))

OR ((ga = 41) AND (# visits>=14 AND # visits<=17))

OR ((ga = 42) AND (# visits>=15 AND # visits<=17))

OR ((ga>=43 AND ga<=45) AND (# visits>=16 AND # visits<=18)));

- Intermediate care level

INDEX3 = 2 if the first visit is in the **first** trimester AND

(((ga>=18 AND ga<=21) AND (# visits>=1 AND # visits<=2))

OR ((ga>=22 AND ga<=25) AND (# visits>=2 AND # visits<=3))

OR ((ga>=26 AND ga<=29) AND (# visits>=2 AND # visits<=4))

OR ((ga>=30 AND ga<=31) AND (# visits>=3 AND # visits<=5))

OR ((ga>=32 AND ga<=33) AND (# visits>=4 AND # visits<=6))

OR ((ga>=34 AND ga<=35) AND (# visits>=5 AND # visits<=7))

OR ((ga = 36) AND (# visits>=5 AND # visits<=8))

OR ((ga = 37) AND (# visits>=6 AND # visits<=9))

OR ((ga = 38) AND (# visits>=7 AND # visits<=10))

OR ((ga = 39) AND (# visits>=7 AND # visits<=11))

OR ((ga = 40) AND (# visits>=8 AND # visits<=12))

OR ((ga = 41) AND (# visits>=8 AND # visits<=13))

OR ((ga = 42) AND (# visits>=9 AND # visits<=14))

OR ((ga>=43 AND ga<=45) AND (# visits>=9 AND # visits<=15)));

OR if the first visit is in the **second** trimester AND

(((ga>=18 AND ga<=21) AND (# visits>=1 AND # visits<=9))

OR ((ga>=22 AND ga<=25) AND (# visits>=2 AND # visits<=10))

OR ((ga>=26 AND ga<=29) AND (# visits>=2 AND # visits<=11))

OR ((ga>=30 AND ga<=31) AND (# visits>=3 AND # visits<=11))

OR ((ga>=32 AND ga<=33) AND (# visits>=4 AND # visits<=12))

OR ((ga>=34 AND ga<=35) AND (# visits>=5 AND # visits<=12))

OR ((ga>=36 AND ga<=37) AND (# visits>=6 AND # visits<=13))

OR ((ga>=38 AND ga<=39) AND (# visits>=7 AND # visits<=14))

OR ((ga==40) AND (# visits>=8 AND # visits<=14))

OR ((ga==41) AND (# visits>=8 AND # visits<=15))

OR ((ga==42) AND (# visits>=9 AND # visits<=15))

OR ((ga>=43 AND ga<=45) AND (# visits>=9 AND # visits<=16)));

- Inadequate care level

INDEX3 = 1 if the first visit is in the **first** trimester AND

(((ga>=22 AND ga<=29) AND (# visits==1))

OR ((ga>=30 AND ga<=31) AND (# visits>=1 AND # visits<=2))

OR ((ga>=32 AND ga<=33) AND (# visits>=1 AND # visits<=3))

OR ((ga>=34 AND ga<=36) AND (# visits>=1 AND # visits<=4))

OR ((ga==37) AND (# visits>=1 AND # visits<=5))

OR ((ga>=38 AND ga<=39) AND (# visits>=1 AND # visits<=6))

OR ((ga>=40 AND ga<=41) AND (# visits>=1 AND # visits<=7))

OR ((ga>=42 AND ga<=45) AND (# visits>=1 AND # visits<=8)));

OR if the first visit is in the **second** trimester AND

(((ga>=22 AND ga<=29) AND (# visits==1))

OR ((ga>=30 AND ga<=31) AND (# visits>=1 AND # visits<=2))

OR ((ga>=32 AND ga<=33) AND (# visits>=1 AND # visits<=3))

OR ((ga>=34 AND ga<=35) AND (# visits>=1 AND # visits<=4))

OR ((ga>=36 AND ga<=37) AND (# visits>=1 AND # visits<=5))

OR ((ga>=38 AND ga<=39) AND (# visits>=1 AND # visits<=6))

OR ((ga>=40 AND ga<=41) AND (# visits>=1 AND # visits<=7))

OR ((ga>=42 AND ga<=45) AND (# visits>=1 AND # visits<=8)));

OR if the first visit is in the **third** trimester AND

(((ga=25) AND (# visits==1 AND # visits<=8))

OR ((ga>=26 AND ga<=31) AND (# visits>=1 AND # visits<=9))

OR ((ga>=32 AND ga<=35) AND (# visits>=1 AND # visits<=10))

OR ((ga>=36 AND ga<=37) AND (# visits>=1 AND # visits<=11))

OR ((ga>=38 AND ga<=40) AND (# visits>=1 AND # visits<=12))

OR ((ga>=41 AND ga<=42) AND (# visits>=1 AND # visits<=13))

OR ((ga>=43 AND ga<=45) AND (# visits>=1 AND # visits<=14)));

- No care

INDEX3 = 0 if # visits = 0
