# Supplementary material for: Prenatal care and child growth and schooling in four low- and medium-income countries
Source: PLoS One. 2017 Feb 3;12(2):e0171299. doi: 10.1371/journal.pone.0171299 (PMC5291430; doi:10.1371/journal.pone.0171299)
Supplement: S1 Table — INDEX1 is the sum of three binary prenatal care variables: ever had prenatal care visits, number of prenatal care visits higher than local medium level and visit in the first trimester. INDEX3 was defined according to the Revised GINDEX as in [50]. (DOCX) [file pone.0171299.s006.docx]

**S1 Table. Cross-classification of two indicators of prenatal care utilization in four birth cohorts (n= 4146)**

|  | **INDEX3** | | | | | |
| --- | --- | --- | --- | --- | --- | --- |
| **INDEX1** | **No visits** | **Inadequate** | **Intermediate** | **Adequate** | **Intensive** | **Total** |
| 0 | 892 | 0 | 0 | 0 | 0 | 892 |
| 1 | 0 | 1061 | 25 | 0 | 0 | 1086 |
| 2 | 0 | 752 | 439 | 0 | 28 | 1219 |
| 3 | 0 | 311 | 507 | 120 | 11 | 949 |
| Total | 892 | 2124 | 971 | 120 | 39 | 4146 |
