# Supplementary material for: Prenatal care and child growth and schooling in four low- and medium-income countries
Source: PLoS One. 2017 Feb 3;12(2):e0171299. doi: 10.1371/journal.pone.0171299 (PMC5291430; doi:10.1371/journal.pone.0171299)
Supplement: S2 Table — The maternal prenatal care utilization index is the sum of three binary prenatal care variables: ever had prenatal care visits, number of prenatal care visits higher than local medium level and visit in the first trimester. *** p value<0.001, ** p value<0.01, * p value<0.05. The models adjusted for controls including maternal schooling, age, height, race, marital status, household composition, wealth, and occupational class. The mediation model (4) also controls for birth weight and the mediation model (5) controls for birth weight and HAZ at 24 mo. Data were analyzed using linear regressions with multiple imputations (20 times) of missing control variables only. 95% confidence intervals are reported in parentheses. (DOCX) [file pone.0171299.s007.docx]

**S2 Table. Associations of maternal prenatal care utilization index with offspring outcomes in four birth cohorts (n=4062)**

|  | **NON-MEDIATION MODEL** | | | **MEDIATION MODEL** | |
| --- | --- | --- | --- | --- | --- |
|  | **(1)** | **(2)** | **(3)** | **(4)** | **(5)** |
| Birth weight | 0 (-0.03 to 0.03) |  |  |  |  |
|  | p = 0.82 |  |  |  |  |
| HAZ at 24 months |  | 0.10* (0.04 to 0.15) |  | 0.09** (0.05 to 0.13) |  |
|  |  | p = 0.01 |  | p = 0 |  |
| Highest attained grade |  |  | 0.26**(0.17 to 0.35) |  | 0.22**(0.12 - 0.33) |
|  |  |  | p = 0.01 |  | p = 0.01 |
