# Supplementary material for: Prenatal care and child growth and schooling in four low- and medium-income countries
Source: PLoS One. 2017 Feb 3;12(2):e0171299. doi: 10.1371/journal.pone.0171299 (PMC5291430; doi:10.1371/journal.pone.0171299)
Supplement: S5 Table — INDEX3 was defined according to the Revised GINDEX in [50]. *** p value<0.001, ** p value<0.01, * p value<0.05. The models adjusted for controls including maternal schooling, age, height, race, marital status, household composition, wealth, and occupational class. The mediation model (4) also controls for birth weight and mediation model (5) controls for birth weight and HAZ at 24 mo. Data were analyzed using linear regressions with multiple imputations (20 times) of missing control variables, gestational age, prenatal care variables and INDEX3 jointly, with variances clustered at site level. 95% confidence intervals are reported in parentheses. (DOCX) [file pone.0171299.s010.docx]

**S5 Table. Pooled analysis of birth and later outcomes with INDEX3 (N=7203)**

|  | **NON-MEDIATION MODELS** | | | **MEDIATION MODELS** | |
| --- | --- | --- | --- | --- | --- |
|  | **(1)** | **(2)** | **(3)** | **(4)** | **(5)** |
|  | **Birth weight** | **HAZ at 24 months** | **Highest attained grade** | **HAZ at 24 months** | **Highest attained grade** |
| Inadequate care | 0.02(-0.05 - 0.08) | 0.03(-0.08 - 0.14) | 0.2(-0.34 - 0.74) | 0.02(-0.10 - 0.14) | 0.19(-0.31 - 0.70) |
|  | P=0.57 | P=0.38 | P=0.28 | P=0.49 | P=0.29 |
| Intermediate care | 0.09**(0.05 - 0.12) | 0.12*(0.00 - 0.25) | 0.25(-0.27 - 0.77) | 0.07(-0.07 - 0.21) | 0.2(-0.30 - 0.71) |
|  | P=0.00 | P=0.05 | P=0.22 | P=0.21 | P=0.28 |
| Adequate care | 0(-0.08 - 0.07) | 0.1(-0.12 - 0.31) | 0.39*(0.14 - 0.63) | 0.1(-0.09 - 0.29) | 0.35*(0.02 - 0.69) |
|  | P=0.88 | P=0.27 | P=0.01 | P=0.22 | P=0.02 |
| Intensive care | 0.07(-0.01 - 0.14) | -0.14(-0.51 - 0.24) | 0.61(-0.28 - 1.49) | -0.18(-0.52 - 0.16) | 0.66(-0.25 - 1.57) |
|  | P=0.15 | P=0.47 | P=0.09 | P=0.31 | P=0.09 |
| F test of joint significance of INDEX3 | 347.2 | 37.03 | 379.7 | 13.84 | 801 |
| prob>F | 0.00025 | 0.00691 | 0.000219 | 0.0282 | 7.15E-05 |
