# Supplementary material for: Prenatal care and child growth and schooling in four low- and medium-income countries
Source: PLoS One. 2017 Feb 3;12(2):e0171299. doi: 10.1371/journal.pone.0171299 (PMC5291430; doi:10.1371/journal.pone.0171299)
Supplement: S6 Table — The sample consists of all the observations without missing prenatal care utilization index in the original sample. The index is the sum of three binary prenatal care variables: ever had prenatal care visits, number of prenatal care visits higher than local medium level and visit in the first trimester. Mother’s schooling is years of completed education. Crowding index denotes the ratio of the number of people over the rooms of the house. Occupational class represents father/mother’s occupation in six rank ordered categories, with unemployed as 0, lowest occupational class as 1, moving up to 5 for the highest class such as professional, technical and commercial. Wealth quintile is the household asset score grouped in quintiles for each country site, running from 1 (poorest) to 5 (wealthiest). The definition of asset is also site specific. Accessibility index to health care facilities is a binary variable with 1 for good access to health services, and defined site specifically: Brazil defined it based on principal component of a few continuous variables in assessing accessibilities; for Guatemala, it is defined based on the distance from home to supplement center where antenatal care is implemented during the study period; for the Philippines, it is defined based on the average travel time to the closest public or private health facility; and for the South Africa, poor accessibility is defined if the mother reported travelling over 45 minutes to her nearest well-baby clinic or if reported no antenatal care. Morbidity at 1st-3rd trimester for Guatemala are the number of types of morbidity (among three types: respiratory disease, diarrhea, fever) during each trimester self-reported by the pregnant mothers to survey enumerator, in contrast to medical records which are more likely endogenous. *** p value<0.001, ** p value<0.01, * p value<0.05. Data were analyzed using linear regressions with multiple imputations (20 times) of missing control variables, with variances clu [file pone.0171299.s011.docx]

**S6 Table. Associations of SES and other household characteristics with prenatal care utilization index in four birth cohorts**

|  | **(1)** | **(2)** | **(3)** | **(4)** | **(5)** |
| --- | --- | --- | --- | --- | --- |
| **Variables** | **Pooled** | **Brazil** | **Guatemala** | **the Philippines** | **South Africa** |
|  |  |  |  |  |  |
| Guatemala | 0.41*** |  |  |  |  |
|  | (0.31 - 0.51) |  |  |  |  |
| the Philippines | -0.72*** |  |  |  |  |
|  | (-0.86 - -0.57) |  |  |  |  |
| Urban Philippines | 0.14* |  |  |  |  |
|  | (0.02 - 0.26) |  |  |  |  |
| South Africa | -0.28 |  |  |  |  |
|  | (-0.88 - 0.32) |  |  |  |  |
| Have flush toilet at home (1=yes) | 0.08 | 0.11 | -0.25 | 0.13 | 0.46 |
|  | (-0.13 - 0.29) | (-0.05 - 0.27) | (-0.69 - 0.19) | (-0.09 - 0.35) | (-0.29 - 1.20) |
| Have good access to water (1=yes) | 0.13 | 0.08 | -0.01 | 0.07 | 0.08 |
|  | (-0.02 - 0.28) | (-0.08 - 0.23) | (-0.50 - 0.49) | (-0.09 - 0.23) | (-0.05 - 0.22) |
| Household crowding index | -0.01* | -0.04 | -0.01 | -0.02 | -0.01 |
|  | (-0.02 - -0.00) | (-0.09 - 0.00) | (-0.06 - 0.04) | (-0.05 - 0.01) | (-0.04 - 0.03) |
| Child dependence ratio | -0.06 | -0.05 | 0.04 | -0.03 | -0.06 |
|  | (-0.14 - 0.02) | (-0.21 - 0.10) | (-0.18 - 0.27) | (-0.15 - 0.09) | (-0.23 - 0.12) |
| Social class (second lowest) | 0.09 | 0.10 | -0.05 | 0.19 | 0.04 |
|  | (-0.02 - 0.19) | (-0.03 - 0.23) | (-0.40 - 0.31) | (-0.01 - 0.38) | (-0.07 - 0.15) |
| Social class (middle) | 0.07 | 0.19 | -0.08 | 0.17* | -0.02 |
|  | (-0.07 - 0.21) | (-0.06 - 0.43) | (-0.44 - 0.28) | (0.01 - 0.33) | (-0.20 - 0.16) |
| Social class (second highest) | 0.23* | 0.36** | 0.20 | 0.26* | 0.29** |
|  | (0.06 - 0.41) | (0.13 - 0.59) | (-0.45 - 0.84) | (0.01 - 0.51) | (0.11 - 0.48) |
| Social class (highest) | 0.24 | 0.20* | 0.48 | 0.36 | 0.43 |
|  | (-0.00 - 0.49) | (0.01 - 0.40) | (-0.65 - 1.61) | (-0.01 - 0.73) | (-0.07 - 0.93) |
| Wealth quintiles (second) | 0.05 | 0.05 | -0.24 | 0.16* | -0.07 |
|  | (-0.15 - 0.26) | (-0.12 - 0.22) | (-0.58 - 0.09) | (0.01 - 0.30) | (-0.24 - 0.10) |
| Wealth quintiles (third) | 0.12 | 0.09 | -0.31 | 0.23*** | -0.05 |
|  | (-0.07 - 0.32) | (-0.11 - 0.29) | (-0.64 - 0.02) | (0.10 - 0.36) | (-0.21 - 0.11) |
| Wealth quintiles (fourth) | 0.13 | 0.07 | -0.27 | 0.34*** | -0.00 |
|  | (-0.19 - 0.46) | (-0.19 - 0.33) | (-0.64 - 0.10) | (0.16 - 0.53) | (-0.17 - 0.17) |
| Wealth quintiles (top) | 0.15 | 0.02 | -0.28 | 0.37*** | 0.08 |
|  | (-0.31 - 0.60) | (-0.23 - 0.27) | (-0.70 - 0.14) | (0.20 - 0.53) | (-0.12 - 0.28) |
| Maternal age | 0.10* | 0.15*** | -0.06 | 0.09** | 0.03 |
|  | (0.00 - 0.19) | (0.08 - 0.21) | (-0.20 - 0.07) | (0.03 - 0.16) | (-0.04 - 0.10) |
| Maternal age squared | -0.00* | -0.00** | 0.00 | -0.00* | -0.00 |
|  | (-0.00 - -0.00) | (-0.00 - -0.00) | (-0.00 - 0.00) | (-0.00 - -0.00) | (-0.00 - 0.00) |
| Maternal schooling | 0.04* | 0.03*** | 0.01 | 0.06*** | -0.01 |
|  | (0.00 - 0.08) | (0.01 - 0.05) | (-0.06 - 0.09) | (0.04 - 0.08) | (-0.03 - 0.01) |
| Maternal height | 0.00 | -0.00 | -0.01 | 0.00 | 0.00 |
|  | (-0.01 - 0.01) | (-0.01 - 0.01) | (-0.03 - 0.02) | (-0.01 - 0.01) | (-0.01 - 0.01) |
| Mother's marital status (1=married) | 0.44 | 0.93*** | 0.66** | 0.38 | 0.09 |
|  | (-0.46 - 1.34) | (0.75 - 1.11) | (0.16 - 1.16) | (-0.32 - 1.08) | (-0.03 - 0.21) |
| Child's sex (1=girl) | -0.03 | 0.03 | 0.17 | -0.12** | -0.03 |
|  | (-0.22 - 0.15) | (-0.08 - 0.13) | (-0.05 - 0.38) | (-0.21 - -0.03) | (-0.12 - 0.07) |
| Child's birth order | -0.14 | -0.21*** | -0.03 | -0.14** | -0.03 |
|  | (-0.29 - 0.00) | (-0.29 - -0.12) | (-0.23 - 0.17) | (-0.22 - -0.05) | (-0.13 - 0.07) |
| The only child (1=yes) | 0.04 | 0.07 | -0.23 | 0.11 | 0.08 |
|  | (-0.03 - 0.12) | (-0.11 - 0.25) | (-0.73 - 0.27) | (-0.07 - 0.28) | (-0.10 - 0.25) |
| Accessibility index to health care facilities | 0.30* | 0.30*** | 0.05 | 0.25*** | 0.90*** |
|  | (0.01 - 0.58) | (0.19 - 0.42) | (-0.17 - 0.27) | (0.11 - 0.39) | (0.73 - 1.08) |
| Urban residents (1=yes) |  |  |  | 0.10 |  |
|  |  |  |  | (-0.03 - 0.22) |  |
| Morbidity at 1^st^ trimester |  |  | 0.31** |  |  |
|  |  |  | (0.12 - 0.49) |  |  |
| Morbidity at 2^nd^ trimester |  |  | 0.30*** |  |  |
|  |  |  | (0.13 - 0.47) |  |  |
| Morbidity at 3^rd^ trimester |  |  | 0.27** |  |  |
|  |  |  | (0.11 - 0.42) |  |  |
| Constant | -0.24 | -1.23 | 2.83 | -0.99 | -0.38 |
|  | (-1.85 - 1.38) | (-2.88 - 0.42) | (-0.87 - 6.54) | (-2.73 - 0.75) | (-2.20 - 1.44) |
|  |  |  |  |  |  |
| N | 4,333 | 1,253 | 471 | 1,870 | 739 |
| F-test | 2457 | 25.64 | 2.994 | 14.79 | 7.106 |
| prob > F | 1.17e-05 | 0 | 4.03e-06 | 0 | 0 |
