# Supplementary material for: Prenatal care and child growth and schooling in four low- and medium-income countries
Source: PLoS One. 2017 Feb 3;12(2):e0171299. doi: 10.1371/journal.pone.0171299 (PMC5291430; doi:10.1371/journal.pone.0171299)
Supplement: S7 Table — Prenatal care utilization index is the sum of three binary prenatal care variables: ever had prenatal care visits, number of prenatal care visits higher than local medium level and visit in the first trimester. *** p value<0.001, ** p value<0.01, * p value<0.05. All the models are adjusted for controls including child’s gender, birth order, whether being the only child at birth, maternal schooling, age, height, race, marital status, household composition, wealth, and occupational class as well as country fixed effects. The mediation model (4) also controls for birth weight and the mediation model (5) controls for birth weight and HAZ at 24 mo. Data were analyzed using linear regressions with multiple imputations (20 times) of missing control variables, gestational age, prenatal care variables and prenatal care utilization jointly, with variances clustered at site level. 95% confidence intervals are reported in parentheses. (DOCX) [file pone.0171299.s012.docx]

**S7 Table. Associations of maternal prenatal care utilization index and all the controls with offspring outcomes in four birth cohorts (n=7203)**

|  | NON-MEDIATION MODELS | | | MEDIATION MODELS | |
| --- | --- | --- | --- | --- | --- |
|  | (1) | (2) | (3) | (4) | (5) |
| VARIABLES | Birth weight | HAZ at 24 months | Highest attained grade | HAZ at 24 months | Highest attained grade |
|  |  |  |  |  |  |
| INDEX3 | 0.01 | 0.09** | 0.26** | 0.09** | 0.23** |
|  | (-0.04 - 0.07) | (0.04 - 0.15) | (0.15 - 0.36) | (0.05 - 0.12) | (0.11 - 0.34) |
|  | p=0.45 | p=0.01 | p=0.00 | p=0.00 | p=0.01 |
| Gestational age | 0.07* | 0.05* | 0.02 | 0 | -0.01 |
|  | (0.01 - 0.14) | (0.01 - 0.08) | (-0.04 - 0.07) | (-0.03 - 0.03) | (-0.06 - 0.04) |
|  | p=0.04 | p=0.02 | p=0.41 | p=0.76 | p=0.70 |
| Guatemala | -0.05 | -1.27*** | -1.91*** | -1.24*** | -1.51** |
|  | (-0.13 - 0.04) | (-1.53 - -1.01) | (-2.35 - -1.47) | (-1.53 - -0.94) | (-1.91 - -1.10) |
|  | p=0.17 | p=0.00 | p=0.00 | p=0.00 | p=0.00 |
| the Philippines | -0.09 | -1.08*** | 1.39** | -1.02*** | 1.74*** |
|  | (-0.22 - 0.04) | (-1.27 - -0.90) | (0.94 - 1.85) | (-1.27 - -0.77) | (1.44 - 2.03) |
|  | p=0.13 | p=0.00 | p=0.00 | p=0.00 | p=0.00 |
| Urban Philippines | 0.04** | 0.02 | 0.35* | -0.01 | 0.34* |
|  | (0.03 - 0.06) | (-0.05 - 0.10) | (0.06 - 0.64) | (-0.08 - 0.07) | (0.07 - 0.60) |
|  | p=0.00 | p=0.40 | p=0.03 | p=0.83 | p=0.03 |
| South Africa | -0.12 | -0.63*** | 0.89* | -0.56*** | 1.10* |
|  | (-0.25 - 0.01) | (-0.76 - -0.51) | (0.15 - 1.63) | (-0.64 - -0.47) | (0.32 - 1.88) |
|  | p=0.07 | p=0.00 | p=0.03 | p=0.00 | p=0.02 |
| Have flush toilet at home (1=yes) | 0.05 | 0.2 | 0.65 | 0.17 | 0.58 |
|  | (-0.02 - 0.12) | (-0.01 - 0.42) | (-0.10 - 1.39) | (-0.09 - 0.43) | (-0.14 - 1.30) |
|  | p=0.11 | p=0.06 | p=0.07 | p=0.13 | p=0.08 |
| Have good access to clean water (1=yes) | 0.02 | 0.08 | 0.26 | 0.06 | 0.23 |
|  | (-0.04 - 0.08) | (-0.09 - 0.25) | (-0.05 - 0.57) | (-0.12 - 0.24) | (-0.06 - 0.52) |
|  | p=0.29 | p=0.24 | p=0.08 | p=0.35 | p=0.08 |
| Household crowding index | 0 | -0.04 | -0.12 | -0.04 | -0.11 |
|  | (-0.01 - 0.01) | (-0.10 - 0.01) | (-0.25 - 0.01) | (-0.10 - 0.01) | (-0.22 - 0.01) |
|  | p=0.70 | p=0.08 | p=0.06 | p=0.08 | p=0.06 |
| Child dependence ratio | -0.04 | -0.18** | -0.21 | -0.15** | -0.15 |
|  | (-0.10 - 0.02) | (-0.23 - -0.13) | (-0.46 - 0.04) | (-0.23 - -0.07) | (-0.41 - 0.11) |
|  | p=0.10 | p=0.00 | p=0.08 | p=0.01 | p=0.17 |
| Social class (second lowest) | 0.01 | 0.07 | 0.29 | 0.07 | 0.26 |
|  | (-0.02 - 0.04) | (-0.14 - 0.28) | (-0.09 - 0.66) | (-0.13 - 0.27) | (-0.05 - 0.58) |
|  | p=0.39 | p=0.35 | p=0.09 | p=0.36 | p=0.08 |
| Social class (middle) | 0.01 | 0.07 | 0.38 | 0.06 | 0.36* |
|  | (-0.05 - 0.07) | (-0.12 - 0.26) | (-0.02 - 0.77) | (-0.13 - 0.25) | (0.01 - 0.70) |
|  | p=0.57 | p=0.34 | p=0.06 | p=0.38 | p=0.05 |
| Social class (second highest) | 0.01 | 0.13 | 0.61 | 0.12 | 0.57 |
|  | (-0.06 - 0.08) | (-0.06 - 0.32) | (-0.35 - 1.57) | (-0.03 - 0.28) | (-0.31 - 1.44) |
|  | p=0.62 | p=0.11 | p=0.14 | p=0.08 | p=0.13 |
| Social class (highest) | 0.03 | 0.16* | 1.11* | 0.14* | 1.06* |
|  | (-0.02 - 0.08) | (0.04 - 0.28) | (0.43 - 1.79) | (0.00 - 0.27) | (0.41 - 1.71) |
|  | p=0.13 | p=0.03 | p=0.01 | p=0.05 | p=0.01 |
| Wealth quintile (second) | 0.01 | 0.09 | 0.12 | 0.08 | 0.09 |
|  | (-0.03 - 0.05) | (-0.01 - 0.19) | (-0.54 - 0.78) | (-0.03 - 0.19) | (-0.54 - 0.73) |
|  | p=0.48 | p=0.07 | p=0.60 | p=0.11 | p=0.67 |
| Wealth quintile (third) | 0.03* | 0.15 | 0.42 | 0.13 | 0.37 |
|  | (0.00 - 0.06) | (-0.06 - 0.36) | (-0.62 - 1.46) | (-0.08 - 0.34) | (-0.60 - 1.35) |
|  | p=0.04 | p=0.11 | p=0.29 | p=0.15 | p=0.31 |
| Wealth quintile (fourth) | 0.02 | 0.15* | 0.42 | 0.13* | 0.37 |
|  | (-0.06 - 0.11) | (0.05 - 0.24) | (-0.28 - 1.12) | (0.00 - 0.26) | (-0.30 - 1.04) |
|  | p=0.44 | p=0.02 | p=0.15 | p=0.05 | p=0.18 |
| Wealth quintile (top) | 0.02 | 0.27** | 0.5 | 0.26** | 0.42 |
|  | (-0.03 - 0.07) | (0.18 - 0.36) | (-0.30 - 1.31) | (0.17 - 0.35) | (-0.35 - 1.18) |
|  | p=0.28 | p=0.00 | p=0.14 | p=0.00 | p=0.18 |
| Maternal age | 0.02* | 0.09** | 0.07 | 0.08** | 0.03 |
|  | (0.01 - 0.04) | (0.07 - 0.12) | (-0.11 - 0.24) | (0.05 - 0.10) | (-0.13 - 0.19) |
|  | p=0.01 | p=0.00 | p=0.31 | p=0.00 | p=0.55 |
| Maternal age squared | -0.00* | -0.00** | 0 | -0.00** | 0 |
|  | (-0.00 - -0.00) | (-0.00 - -0.00) | (-0.00 - 0.00) | (-0.00 - -0.00) | (-0.00 - 0.00) |
|  | p=0.02 | p=0.00 | p=0.62 | p=0.01 | p=0.99 |
| Maternal schooling | 0.01 | 0.02 | 0.22** | 0.02 | 0.21** |
|  | (-0.00 - 0.01) | (-0.00 - 0.05) | (0.11 - 0.33) | (-0.01 - 0.05) | (0.11 - 0.32) |
|  | p=0.20 | p=0.07 | p=0.01 | p=0.10 | p=0.01 |
| Maternal height | 0.01*** | 0.06*** | 0.03** | 0.05*** | 0.01 |
|  | (0.01 - 0.02) | (0.05 - 0.07) | (0.02 - 0.04) | (0.05 - 0.06) | (-0.00 - 0.02) |
|  | p=0.00 | p=0.00 | p=0.00 | p=0.00 | p=0.06 |
| Mother's marital status (1=married) | -0.01 | 0.09* | 0.12 | 0.1 | 0.09 |
|  | (-0.17 - 0.15) | (0.01 - 0.17) | (-0.65 - 0.89) | (-0.01 - 0.20) | (-0.65 - 0.83) |
|  | p==0.85 | p=0.03 | p=0.66 | p=0.06 | p=0.72 |
| Child's sex (1=girl) | -0.11* | 0.15** | 0.82* | 0.22** | 0.78* |
|  | (-0.18 - -0.03) | (0.10 - 0.20) | (0.28 - 1.35) | (0.13 - 0.31) | (0.26 - 1.30) |
|  | p=0.02 | p=0.00 | p=0.02 | p=0.00 | p=0.02 |
| Child's birth order | 0.03* | -0.15** | -0.29 | -0.17** | -0.25 |
|  | (0.01 - 0.04) | (-0.19 - -0.10) | (-0.65 - 0.06) | (-0.22 - -0.12) | (-0.60 - 0.10) |
|  | p==0.01 | p=0.00 | p=0.08 | p=0.00 | p=0.11 |
| The only child (1=yes) | -0.07* | -0.01 | -0.09 | 0.04 | -0.08 |
|  | (-0.12 - -0.02) | (-0.05 - 0.03) | (-0.44 - 0.27) | (-0.01 - 0.09) | (-0.43 - 0.28) |
|  | p=0.02 | p=0.68 | p=0.50 | p=0.07 | p=0.54 |
| Birth weight |  |  |  | 0.66** | 0.1 |
|  |  |  |  | (0.48 - 0.83) | (-0.06 - 0.27) |
|  |  |  |  | p=0.00 | p=0.14 |
| Height-for-age z score at 24 months |  |  |  |  | 0.31* |
|  |  |  |  |  | (0.14 - 0.49) |
|  |  |  |  |  | p=0.01 |
| Constant | -2.07 | -14.23*** | -0.47 | -12.87*** | 4.19* |
|  | (-4.73 - 0.60) | (-16.32 - -12.14) | (-6.65 - 5.71) | (-14.28 - -11.47) | (0.41 - 7.96) |
|  |  |  |  |  |  |
| Observations | 7,203 | 7,203 | 7,203 | 7,203 | 7,203 |
| F-test | 810.3 | 1532 | 4212 | 1546 | 4242 |
| prob > F | 6.21E-05 | 2.39E-05 | 5.24E-06 | 2.36E-05 | 5.19E-06 |
