# Supplementary material for: Prenatal care and child growth and schooling in four low- and medium-income countries
Source: PLoS One. 2017 Feb 3;12(2):e0171299. doi: 10.1371/journal.pone.0171299 (PMC5291430; doi:10.1371/journal.pone.0171299)
Supplement: S8 Table — For Guatemala sample, there is no enough variation in breastfeeding incidence for estimation (only 1 out of 489 observations having no breastfeeding). Data were analyzed using probit model (for breastfeeding incidence) and linear regression (for breastfeeding duration) with multiple imputations (20 times) of missing control variables, gestational age and prenatal care utilization index jointly, with variances clustered at site level. 95% confidence intervals are reported in parentheses. (DOCX) [file pone.0171299.s013.docx]

**S8 Table. Associations between prenatal care utilization index and breastfeeding incidence and duration**

|  | **Brazil** | **Guatemala** | **Philippines** | **South Africa** | **Pooled** |
| --- | --- | --- | --- | --- | --- |
| *N* | 3633 | 467 | 1935 | 1043 | 7078 |
| Breastfeeding incidence | 0.08*  (-0.01 - 0.17) | . | 0.04  (-0.08 - 0.15) | -0.01  (-0.24 - 0.23) | 0.05  (-0.02 - 0.12) |
|  | p =0.07 |  | p =0.53 | p=0.96 | p =0.15 |
| Breastfeeding duration | -0.03  (-0.13 - 0.08) | -0.01  (-0.08 - 0.06) | -0.14***  (-0.24 - -0.04) | -0.06  (-0.31 – 0.19) | -0.09  (-0.40 - 0.21) |
|  | p =0.61 | p =0.80 | p=0.005 | p =0.64 | p =0.31 |
